# Validación de una Intervención para Fortalecer el Autocuidado en Estudiantes de Enfermería*

**DOI:** 10.15649/cuidarte.2540

**Published:** 2023-09-03

**Authors:** Moises Alfonso Bravo-Gomez, Leidy Yemile Vargas-Rodríguez, Marcela Arenas-Cardenas, Mauricio Lopez-Solano, Raquel Rivera-Carvajal

**Affiliations:** 1 . Universidad de Santander. Facultad de Ciencias Médicas y de la Salud Instituto de Investigación Masira. Bucaramanga, Colombia. Email: mo.bravo@mail.udes.edu.co Universidad de Santander Universidad de Santander Facultad de Ciencias Médicas y de la Salud Instituto de Investigación Masira Bucaramanga Colombia mo.bravo@mail.udes.edu.co; 2 . Universidad de Boyacá, Tunja, Colombia. Email: leiyemvargas@uniboyaca.edu.co Universidad de Boyacá Universidad de Boyacá Tunja Colombia leiyemvargas@uniboyaca.edu.co; 3 . Universidad de Boyacá, Tunja, Colombia. E-mail: ymarenas@uniboyaca.edu.co Universidad de Boyacá Universidad de Boyacá Tunja Colombia ymarenas@uniboyaca.edu.co; 4 . Universidad de Santander, Bucaramanga, Colombia. Email: maurdopezsdano@gmailjcom Universidad de Santander Universidad de Santander Bucaramanga Colombia maurdopezsdano@gmailjcom; 5 . Universidad de Santander. Facultad de Ciencias Médicas y de la Salud Instituto de Investigación Masira. Bucaramanga, Colombia. E-mail: raq.rivera@mail.udes.edu.co Universidad de Santander Universidad de Santander Facultad de Ciencias Médicas y de la Salud Instituto de Investigación Masira Bucaramanga Colombia raq.rivera@mail.udes.edu.co

**Keywords:** Estudio de Validación, Educación en Salud, Autocuidado, Estudiantes de Enfermería, Validation Study, Health Education, Self-care, Students, Nursing, Estudo de validagáo, Educagáo em Saúde, Autocuidado, Estudantes de enfermagem

## Abstract

**Introducción:**

El autocuidado es uno de los conceptos a fortalecer en futuros profesionales de enfermería desde la formación centrada en el aprendiente.

**Objetivo:**

Estimar la validación de una estrategia educativa a través del juicio de expertos y de la población objetivo.

**Materiales y Métodos:**

La investigación se desarrolla bajo un enfoque mixto cual-cuant en paralelo convergente secuencial. En la fase cualitativa 6 estudiantes, un análisis lexicometrico de similitud y fenomelogico hermeneutico. La fase cuantitativa con juicio de 10 expertos, se calculo IVC y media artimetica para pertinencia, coherencia, relevancia y claridad, para concordancia se utilizó la prueba de Brennan and Prediger.

**Resultados:**

Se identifica las relaciones entre la inducción a la acción y la introspección de conductas de autocuidado. El IVC general fue 0.96, una media de 3.7±0.4. En concordancia el item “objetivos especificos” fue el que presento valor p=0.054, coef=0.37, en claridad presenta los menores puntajes en la media (3.4±0.8) e IVC (0.8), los restantes items presentan valor p>0.05

**Discusión:**

En la validación por parte de los jueces de la intervención educativa, se obtuvo un índice de validez de contenido con valores superiores a lo deseable, lo cual es similar a lo reportado por otros autores donde se obtuvo una valoración de la información como suficiente y necesaria.

**Conclusion:**

Se considera que la intervención educativa cuenta con los criterios de validación de contenido tanto por jueces expertos como de la población objetivo, manifestado por la reflexión hacia la transformación de algunos comportamientos de autocuidado en los estudiantes de enfermería.

## Abstract (en)

**Introduction:**

Self-care is one of the concepts to be strengthened in future nurses from a learner centered education perspective.

**Objective:**

To estimate the validation of an educational strategy through both experts and the target population judgment.

**Materials and Methods:**

The research followed a convergent parallel sequential mixed qualitative-quantitative approach. In the qualitative phase, analysis of lexical similarities and hermeneutic phenomenological analysis was carried out with six students. The quantitative phase included opinions from ten experts; CVI and arithmetic mean were calculated to assess pertinence, coherence, relevance, and clarity. The Brennan-Prediger coefficient was used for measuring agreement.

**Results:**

Relationships between action inducement and introspection of self-care behaviors are identified. Overall CVI was 0.96, with a mean of 3.7±0.4. Regarding agreement, the item ‘specific objectives' was the one that showed a p-value of 0.054 and a coefficient of 0.37. Concerning clarity, the lowest mean (3.4±0.8) and CVI (0.8) values were observed. The remaining items have a p-value >0.05.

**Discussion:**

In validating the educational intervention by the experts, a content validity index was obtained with values higher than desirable. This result is similar to that reported by other authors whose information was rated as sufficient and necessary.

**Conclusion:**

The educational intervention has the criteria for content validity according to both experts and the target population, as manifested by the reflection on the transformation of some self-care behaviors in nursing students.

## Resumo

**Introdujo:**

O autocuidado é um dos conceitos a reforjar nos futuros profissionais de enfermagem a partir da formagáo centrada no aprendente.

**Objetivo:**

Estimar a validagáo de uma estratégia educacional por meio do julgamento de especialistas e da populagáo-alvo.

**Materiais e Métodos:**

A pesquisa é realizada sob uma abordagem qualitativa e quantitativa, mista em paralelo convergente sequencial. Na fase qualitativa 6 alunos, uma análise lexicométrica de similaridade e fenomenológica hermenéutica. A fase quantitativa com julgamento de 10 especialistas, IVC e média aritmética foram calculados para relevancia, coeréncia, relevancia e clareza, para concordancia foi utilizado o teste de Brennan e Prediger.

**Resultados:**

Identificam-se as relagóes entre a indugáo a agáo e a introspegáo dos comportamentos de autocuidado. O IVC geral foi de 0,96, com média de 3,7 ±0,4. Nesse sentido, o item " objetivos específicos " foi o que apresentou valor p=0,054, coef =0,37, claramente apresenta as menores pontuagóes na média (3,4 ±0,8) e IVC (0,8), os demais itens apresentam valor p>0,05

**Discussao:**

Na validagáo pelos juízes da intervengáo educacional, obteve-se um índice de validade de conteúdo com valores acima do desejável, o que é semelhante ao relatado por outros autores onde foi obtida uma avaliagáo da informagáo como suficiente e necessária.

**Conclusao:**

Considera-se que a intervengáo educativa tem os critérios de validagáo de conteúdo tanto por juízes especialistas como pela populagáo-alvo, manifestada pela reflexáo para a transformagáo de alguns comportamentos de autocuidado em estudantes de enfermagem.

## Introducción

El cuidado es la razón de ser de los futuros profesionales de enfermería y sus capacidades de autocuidado estan relacionadas con la capacidades para cuidar a los demás, es así, como la formación de estos nuevos enfermeros y enfermeras debe suponer el fortalecimiento de la agencia de autocuidado de sus estudiantes en cada uno de los niveles de preparación. También, es evidente que los aprendientes universitarios poseen algunas conductas inadecuadas de autocuidado debido a diferentes factores de tipo académico, familiares, sociales, biologicos; los cuales pueden desencadenar un mayor riesgo de llegar a padecer alguna Enfermedad Crónica No Transmisible-ECNT al no ser modificados^1^^-^^5^

Es importante precisar, que algunas de estas conductas inadecuadedas en la agencia de autocuidado presentadas en los estudiantes estan relacionadas con: capacidades para descansar y tener un sueño reparador, hábitos nutricionales saludables, disponer de tiempo para cuidarse por ocupaciones académicas, solicitar ayuda/asesoría en el tratamiento farmacológico y realizar ejercicio o tener una actividad física regular^2^^,^^4^^,^^6^^-^^8^.

Por tal razón, los programas de enfermería de la Universidad de Santader-UDES y de la Universidad de Boyacá-UB proposieron una intervención educativa denominada “Hacia el cuidado de la vida” para mejorar estas capacidades operativas en sus estudiantes con base en los postulados de Dorothea Oream, el Aprendizaje Basado en Problemas-ABP, el aprendizaje reflexivo y la mediación pedagogica, propuesta financiada por la Convocatoria Interna Focalizada de Proyectos de Investigación y Desarrollo Tecnológico 2019 de la UDES.

Dentro del proyecto propuesto, destacaba la validación de la intervención educativa tanto por jueces expertos, como por la población objetivo desde la visión de poder entender la utilidad y prácticidad de dicha intervención^9^. En tal sentido, el grupo investigador reconocio la validación como: “la investigación que se realiza con los representantes de un grupo de personas a los cuales va dirigido un material específico, la finalidad es que ellos opinen sobre los instrumentos antes de que éstos ingresen a la etapa de manufactura”^10^, donde se incluye “la validez de expertos o face validity, la cual se refiere al grado en que aparentemente un instrumento de medición mide la variable en cuestión, de acuerdo con “voces calificadas”. Se encuentra vinculada a la validez de contenido y, de hecho, se consideró por muchos años como parte de ésta. Hoy se concibe como un tipo de evidencia distinta^11^. Con base en lo anterior, el objetivo de la investigación fue Estimar la validación de la estrategia educativa “Hacia el cuidado de la vida” a través del juicio de expertos y de su población objetivo.

## Materiales y Métodos

**Figura 1:**
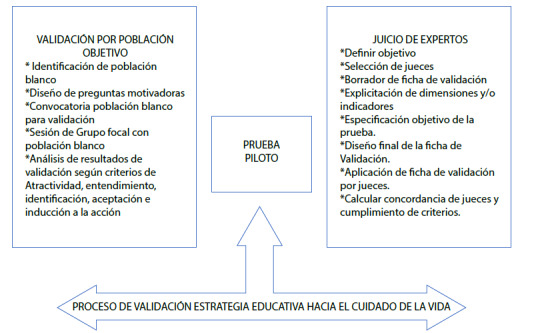
Proceso de validación

Estudio de validación de una intervención educativa con enfoque mixto cual-cuant en paralelo convergente secuencial siguiendo lo propuesto por Creswell^12^ y las recomendaciones de Echer^9^ para intervenciones educativas en salud. La investigación se realizó en el programa de enfermería de la UDES-Sede Bucaramanga-Colombia en el periodo comprendido de febrero a mayo del 2020. En tal sentido, se toma como base las metodologías de juicio de expertos según Escobar^13^ en la fase cuantitativa y el análisis fenomenologico hermeneutico de Fuster^14^ para la fase cualitativa (ver Figura 1).

*Creación propia con base en los postulados de* Escobar^13^*y Ziemendorff & Krause*^10^

### Fase cualitativa o con población objetivo

Durante la fase cualitativa se identificarón los potenciales participantes, se diseñaron preguntas motivadoras con el objetivo de valorar los criterios de atractividad, entendimiento, identificación, aceptación e inducción a la acción propuestos por Ziemendorff & Krause ^10^ para la validación de material educativo (ver Cuadro 1), y como técnica de recolección de datos fue empleada el grupo focal siguiendo las suguerencias de Escobar y Bonilla^15^.

**Cuadro 1:** Criterios de validación de la Intervención educativa “Hacia el cuidado de la vida”.

Criterio	Definición	Pregunta Orientadora
Atractividad	Ayudar a que la intervención educativa (IE) sea apreciada, despierte el ínteres y llame la atención para que ella misma sea percibida.	1. Que le llamo más la atención o motivo de la IE
		2. Los materiales utilizados en la IE como le parecieron
		3. Que le motivo durante el desarrollo de la IE
Entendimiento	Asegurar que la IE sea entendi da. Esta es la finalidad principal de la validación.	4.Que puede decir de las formas, modos y contenidos de la IE ... cómo le parecieron.
		5.Consideran que ese material educativo fue adecuado o fue difícil de entender
Identificación	El grupo se tiene que identificar con la IE por sus personajes, contexto y problemas.	6.En una palabra como define el autocuidado
		7.En su opinión recomendaría participar de la IE a otros estu diantes
Aceptación	Las ideas y propuestas de la IE se encuentran acorde al estudiante y estan al alcance para que ellos vean factible y cumplan con el cambio.	8.Como ven ustedes la posibi lidad de poner en práctica lo aprendido durante el desarrollo de la IE.
Inducción a la acción	Ayudar a la población blanco a cambiar sus comportamientos en la manera deseada.	9.Puede comentarnos alguna experiencia donde considere que la IE le ayudo a tomar decisiones

*Fuente: Creación propia basado en la teoría de Ziemendorff & Krause*^10^

Para la prueba piloto se tomó el grupo de estudiantes de primer semestre de enfermería con quienes se desarrollo la intervención educativa “Hacia el cuidado de la vida” en el periodo de agosto a noviembre del 2019 durante 16 semanas, con encuentros semanales de 1 hora para desarrollar las 4 unidades tematicas (autocuidado mental y espiritual, estilos de vida saludable, autocuidado corporal y autocuidado social) utilizando la metodologia del ABP según los postulados de Guillamet^16^.

El grupo focal se programo con 8 días de anticipación, citando personalmente a los estudiantes. La reunión se cumplió en un salón privado y sin ruidos para favorecer la confidencialidad, fue moderado por los autores de la investigación con experiencia en estudios cualitativos y formación de maestria en enfermería y educación. Estos moderadores presentaron el propósito del estudio a los 11 participantes y posterior a la aceptación verbal firmaron el consentimiento informado 5 estudiantes que decidieron formar parte del grupo focal. El consentimiento informado incluia la posibilidad de grabar audio, lo cual se realizó y la duración de la sesión fue de 53 minutos.

La presente investigación fue aprobada por el comité de bioética de la UDES quien avalo que el estudio cumplia con la declaración de Helsinki y la resolución 8430/93 que establece las normas para la investigación en salud en Colombia al clasificarla en investigación sin riesgo.

El relato experiencial narrativo se realizó utilizando el análisis textual con el programa Iramuteq de la siguiente manera:

1. *Se analizaron las percepciones de los estudiantes respecto de la estrategia educativa “Hacia el cuidado de la vida”, donde dos investigadores leyeron el texto transcrito y las notas de campo posterior a realizar el grupo focal.*
2. Realización de codificaciones correspondientes de los discursos de los estudiantes (ejemplo: **** *Participante_1) en formato UTF 8 - All languages.
3. *A continuación, se analizaron en el software IRAMUTEQ (Interface de R pour les Analyses Multidimensionnelles de Textes et de Questionnaires); incluyendo:*

- *Consignación de los datos con doble digitación y cotejo con el audio.*
- *Transcripción y conformación del corpus textual.*
- *Análisis lexicométrico utilizando el software IRAMUTEQ identificando la repetición y sus relaciones (árbol de conceptos).*

1. *Interpretación de resultados utilizando los criterios de Atractividad, entendimiento, identificación, aceptación e inducción a la acción.*

### Fase cuantitativa o de validación técnica

En el proceso de validacion se consultó a jueces expertos, durante el periodo de febrero a mayo del 2020. Se utilizó un instrumento, donde además de características sociodemográficas y ocupacionales de los jueces se solicitaba que emitieran un concepto de pertinencia, coherencia, relevancia y claridad de los contenidos de la estrategia educativa, donde asignaban un puntaje de 1 a 4, en escala Likert, donde 1 consideraban no cumple con el criterio, 2 es bajo nivel, 3 en moderado nivel y 4 alto nivel de cumplimiento.^13^ El análisis estadístico de las caracteriscas de los jueces se describen utilizando frecuencias relativas y absolutas para las variables categóricas; para las numéricas se presenta la media y desviación estándar, se verifico que presentaran distribucion normal mediante la prueba de Shapiro Francia. El IVC (Indice de Validez de Contenido) se calculó tomando como criterio las respuestas en las categorías 3 y 4 como las aceptables y se dividió por el número de jueces consultados para cada una de los ítems de la encuesta y los conceptos de pertinencia, coherencia, relevancia y claridad. Una puntación superior del IVC a 0,78 se considera deseable^17^.

Para cada uno de los ítems y sus componentes (justificación, objetivos, fundamentación teórica, metodología, consideraciones éticas y referencias) también se calculó el promedio y la desviación estándar de los puntajes asignados por los jueces y la conconcordancia entre ellos, esto último utilizando la prueba estadística de Brennan and Prediger , la concordancia se considerada pobre cuando los puntajes son < 0.00, bajo entre 0.00-0.20, justo 0.21 - 0.40, moderado 0.41- 0.60, sustancial de 0.61 - 0.80 y casi perfecto de 0.81 - 1.00. Se utilizo el programa estadístico Stata V 12 y Excel^18^.

La selección de los jueces cumplió las normas del muestreo por conveniencia y los siguientes criterios de inclusión: Jueces expertos con experiencia profesional en investigación, docencia, enfermería, salud pública, entre otros; experiencia como juez experto en al menos 1 estudio de validación y formación académica mínimo posgrado de tipo maestría. Los criterios de exclusión fueron: Autores de la presente investigación y administrativos que tengan relación con el proyecto. El tiempo de recolección de la información fue de 60 días y el abordaje a los expertos se realizo personalmente a traves de un directorio conformado por los investigadores explicandoles el objetivo del estudio y el cuestionario. Una vez asentía, firmaba la carta de aceptación como juez experto y se entregaba el material de forma impresa o virtual según su conveniencia. Para calcular el número necesario de jueces se tiene en cuenta la teoria de Polit, et al^19^ al recomendar un rango entre 8 a 12 expertos. Los datos de la presente investigacion estan disponibles en Mendeley data^20^.

## Resultados

### Fase cualitativa validación por población objetivo

El corpus textual analizado permitió identificar las frecuencias de las unidades semánticas compuestas por las formas activas o palabras más repetidas: Decir (34v), hacer (19v), pasar (13v), mucho (12v), momento (11v), saber (11v), persona (10v), compañero (9v), conocer (9v), salud (8v), entender (6v), cuidar (5v), entre otras; y dentro de las formas suplementarias sobresalieron: poder (28v), ser (48v) y nosotros (14v).

**Figura 2:**
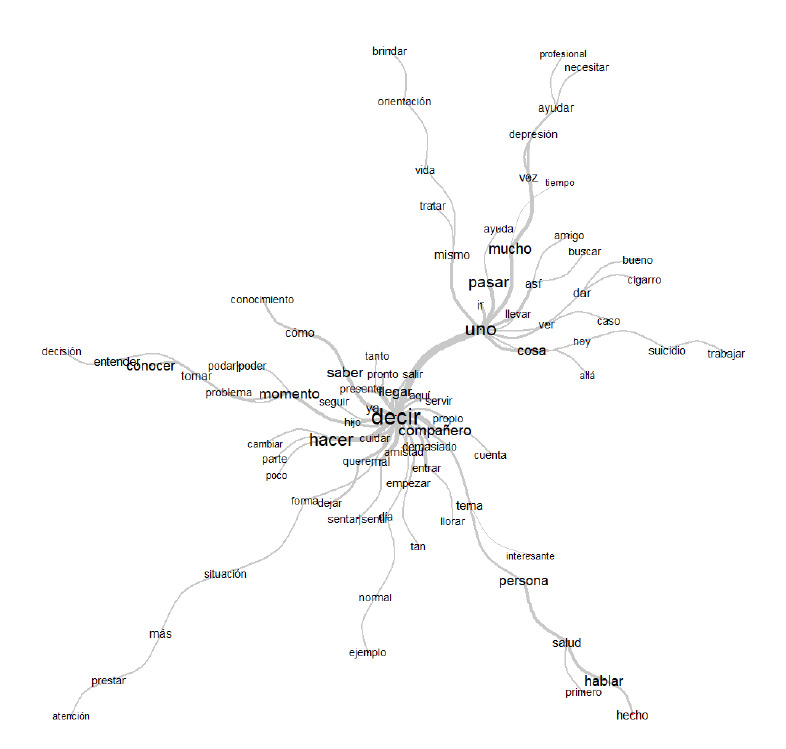
Análisis de similitud: Relaciones entre las formas de las clases Fuente: Creación propia software Iramuteq.

Ahora bien, continuando con el análisis lexicométrico de similitud a través de las palabras más frecuentes de los estudiantes en su discurso (ver Figura 2), se puede inducir un dialogo desde su subjetividad con decir, como también una postura intra e interpersonal con uno, para llegar a hacer o pasar de la teoría a la acción del cuidar, tanto de la persona, como del compañero en un momento o problema basándose en el conocer y el entender. Es importante señalar que los participantes logran reconocer la importancia del bienestar espiritual y mental como unidad temática de la estrategia educativa para prevenir el suicidio y la depresión.

**Cuadro 2:** Correspondencia entre criterios de validación y las Unidades de Contexto Elementales - UCEs

Criterio	Preguntas	UCEs
Atractividad	¿Qué le llamo más la atención o motivo de la IE?	“A mí me pareció una actividad muy interesante, ya que logramos interactuar con el ambiente, como poder estudiar, como aprovechar el campo universitario que tenemos, también me pareció muy interesante saber sobre cómo poder alimentarnos para tener un estilo de vida saludable” (P1)
	¿Los materiales utilizados en la IE como le parecieron?	“De hecho fueron muy adecuados porque si hablamos del tiempo muy pocos teníamos como la posibilidad, porque por ejemplo algunos trabajaban o algunos tenían mucho estrés de la universidad, el tiempo fue adecuado fue un tiempo que se manejó de acuerdo a las necesidades y las posiciones en las que estábamos en ese momento” (P4)
	¿Que le motivo durante el desarrollo de la IE?	“Exacto, pienso que se buscó la estrategia de que todos pudiéramos participar y pudiéramos colocar de nuestra parte para llevar a cabo este tema” (P1)
		“Total, es un tema que es demasiado interesante porque de hecho lo que más se escucha hoy en día es el suicidio y fue uno de los temas que se trataron durante este proyecto, se ve demasiado y de hecho es uno de los ámbitos en los que más hay que trabajarle y que muy poco de pronto se estaba haciendo anteriormente” (P2)
Entendimiento	¿Que puede decir de las formas, modos y contenidos de la IE … cómo le parecieron….fue fácil de entender?	“El autocuidado con uno mismo tanto mental y espiritualmente las creencias que tengamos personas con respecto a eso para poder seguir adelante y poder tener una salud vital a futuro tanto como presente y poderlos como decirlo así enfocar a las personas que tengo yo a mi alrededor tanto hacia mi familia como compañeros y las personas son las que yo me rodeo brindándoles esa incitación u orientación a que tengan una vida saludable” (P3)
	¿Consideran que ese material educativo fue adecuado o fue difícil de entender?	“De hecho si somos un grupo muy pequeño y naturalmente el espacio se ha prestado como para que sea más entendible porque se usaron videos, reflexiones de una forma u otra como que digamos nos conectara al tema porque si le preguntamos a los compañeros yo creo todos tuvimos conexión porque de hecho es un tema que nos preocupa a todos podemos tener familiares amigos hijos” (P2)
		“Fue bastante explicito”(P3) “Fue un documento que nos dio a entender lo que necesitamos”(P5)
		“Fueron cosas de la vida real”(P4)
Identificación	¿En una palabra, como define el autocuidado?	“Quererse así mismo”(P4)
	¿En su opinión, recomendaría participar de la IE a otros estudiantes?	“Amarse”(P2)
		“Valorarse”(P5)
		“Tenerse amor propio”(P3)
		“Aceptarse”(P1)
		“Si, desde primer semestre”(P5)
		“Total, primero estamos hablando de salud, cuando nosotros somos enfermeros no solo hablamos de salud, incluimos la parte física y emocional de las personas”(P2)
		“Si, porque desde el momento que conocemos estos problemas que se vienen presentando nosotros como estudiantes de salud podemos como interiorizar ese tema y tomarlo desde una perspectiva muy única de cada persona al momento”(P5)
		“Entonces con nuestros propios compañeros y con nosotros mismos empezamos a identificar como unas necesidades y cuales conductas podemos tener nosotros mismos y conocerlas y tratar sobre ellas mismas”(P2)
Aceptación	¿ Como ven ustedes la posibilidad de poner en práctica lo aprendido durante el desarrollo de la IE?	“Una de las cosas que aprendimos también cuando hablamos de salud o cuidado y de la alimentación sino también de salud espiritual como seres humanos, como debemos nosotros llenar esa parte que muchas veces traemos vacía, haciendo momentos de relajación” (P2)
		“A no dejarnos llevar porque una de las cosas que veíamos en un caso era que muchas veces las otras amistades influían mucho en la drogadicción en cosas que se puede ver uno inmerso en un adolescente”(P4)
		“También sería el poder entender el lugar del otro la situación que está pasando y poder de una forma brindarle unas asesorías o un acompañamiento, si lo necesita, brindarle información que el necesite para que en lo que esté pasando pueda tener alguien con quien hablar, ayuda profesional, hay profesionales que te pueden ayudar a solucionar este problema y poder brindarle una información”(P3)
Inducción a la acción	¿Puede comentarnos alguna experiencia donde considere que la IE le ayudo a tomar decisiones?	“Bueno como todos saben que yo no soy de aquí siempre he sido muy apegada a mi mamá y a mi papá nunca me había separado tan lejos, entonces pues si me ha dado muy duro porque no doy para estar sola, no me gusta la soledad entonces como que siempre busco estar con alguien, cuando estoy sola entro como que en depresión a llorar y llorar (…) pues estaba sola no habían llegado todos los pensionados y había una muchacha y se me acerco y le dije yo quiero salir yo me siento mal aquí encerrada, salimos y compramos tres cervezas cada una y yo compre una cajita de cigarrillo para fumar, y no se profe que me paso (…) llame a mi compañera-y le dije- me siento mal me dijo esto y esto y esto y pues yo sabía que había obrado mal (…) y como que dije que yo tengo que dejar de hacer eso porque pues lo que hago es como que uno pierde amistades vas perdiendo poco a poco las cosas que realmente quiere y pues desde ese entonces mi compañera aquí presente sabe que yo dije que tengo que cambiar, voy a cambiar y saber lo que estoy haciendo”(P1)
		“A mi hermano le diagnosticaron hace un año con VIH-SIDA positivo y él ha entrado como en esa crisis que ahorita está demasiado mal no se ha querido cuidar porque le mandan esos medicamentos que le ayuda a subir las defensas y no los consume. Se fue para el campo y yo una de las cosas que le he dicho a él y que me ha aportado demasiado el área que digamos este proyecto que abordamos en algún momento ha sido como tener la delicadeza y saber hablar con él y decirle es que estas mal y aparte tienes un diagnóstico y no te estas cuidando, primero hay que tener amor propio para uno y segundo para los demás porque si no te vas a cuidar (…) Entonces el hecho de tener haber tenido un conocimiento no un conocimiento como tal si no un acercamiento a la salud de cómo hablarles a las personas, también está en una depresión ahorita que la sigue teniendo porque él no quiere cuidarse entonces está en una etapa que uno dice que ya se está dejando llevar no quiere cuidarse, que pase lo que tenga que pasar entonces me ha servido bastante para entender y explicarle como a él las cosas que pueden pasar y que pues la vida no solo se trata de un diagnostico va más allá de esto, va más allá de un cuidado personal de un autocuidado de uno mismo”(P2).

Siguiendo con el ánalisis hermeneutico, se presentan las apreciaciones de los educandos hacia los criterios de de atractividad, entendimiento, identificación, aceptación e inducción a la acción donde podemos encontrar las concordancias que soportarón la validación por la población blanco al destacar la tranferencia del aprendizaje a la experiencia de vida en situaciones individuales y familiares (ver Cuadro 2), evidenciadas en la recolección de anécdotas, la entrevista grupal y las experiencias narradas^10^^,^^14^.

### Fase cuantitativa validación por jueces expertos

Como puede observarse en la Tabla 1 la mayoría de los jueces fueron de género femenino (80,0%) y casados (50%). En su formación académica predomina la maestría (90,0%) y en promedio más de la mitad de los jueces expertos tienen experiencia investigativa, docente y administrativa.

**Tabla 1:** Caracterización de los jueces

VARIABLE	X± DS o % (n) (10)
Edad	44,12 ± 10,63
Sexo	
Masculino	20,00 (2)
Femenino	80,00 (8)
Estado civil	
Soltero	10,00 (1)
Casados	50,00 (5)
Unión libre	20,00 (2)
Separados/Divorciado	20,00 (2)
d>'!' https://dx.doi.org/10.15649/cuidarte.2540	Revista Cuidarte Mayo-Agosto 2023; 14(2): e2540
VARIABLE	X± DS o % (n) (10)
Formación académica	
Magister	90,00 (9)
Doctorado	10,00 (1)
Área de conocimiento	
Investigación	60,00 (6)
Salud pública	30,00 (3)
Docencia	70,00 (7)
Asistencial y/o administrativa	80,00 (8)
Otro	10,00 (1)
Experiencia Profesional Años	18,13 ± 8,51

*Abreviaturas:* X*: media aritmética; DE: Desviación Estándar; % (n): Porcentaje (numero absoluto)*

En la Tabla 2 se aprecia de manera especifica que el valor mínimo del IVC de la intervención educativa “Hacia el cuidado de la vida” en los ítems valorados y sus criterios fue de 0,8 y un valor máximo de 1,0; mientras que, en su apreciación general encontramos un valor mínimo del IVC en 0,93 y un maximo de 0,98; valores que evidencias un valoración superior a lo deseable. También se puede evidenciar la concordancia de los jueces que oscilo entre las categorias de justo (0,37 a 0,54) con 6 items a saber: justificación, objetivos: general y específicos, fundamentación teorica: Autocuidado Dorothea Orem, metodología: recursos didácticos y pedagógicos, consideraciones éticas); y sustancial (0,62 a 0,80) con los 7 ítems restantes.

**Tabla 2:** IVC y promedios según los ítems y criterios de la intervención educativa

Ítems	Pertinencia		Coherencia		Relevancia		Claridad		Total		Concordancia	
	IVC	X±DE	IVC	X ±DE	IVC	X ±DE	IVC	X ±DE	IVC	X ±DE	Coef	P valor
Justificación	1,00	3,7±0,4	0,80	3,6±0,8	1,00	3,8±0.4	0,80	3,5±0,8	0,90	3,6±0,6	0,43	0,004
Objetivos												
General	1,00	3,9±0.3	0,90	3,6±0,6	0,90	3,7±0,6	0,90	3,6±0,6	0,90	3,7±0,5	0,46	0,018
Específicos	0,90	3,6±0,6	1,00	3,6±0,4	1,00	3,9±0,3	0,80	3,4±0,8	0,90	3,6±0,5	0,37	0,054
Fundamentación teórica												
Autocuidado según Dorothea Orem	1,00	3,7±0,4	1,00	3,8±0,4	1,00	3,8±0,4	1,00	3,9±0,3	1,00	3,8±0,3	0,54	0,005
Aprendizaje Basado en Problemas	1,00	3,9±0,3	1,00	3,9±0,3	1,00	3,9±0,3	1,00	3,8±0,4	1,00	3,8±0,3	0,68	0,001
Aprendizaje reflexivo-experiencial	0,90	3,6±0,6	0,90	3,8±0,6	1,00	3,9±0,3	1,00	3,9±0,3	0,900	3,8±0,4	0,62	0,009
Técnica de mediación pedagógica	1,00	4,0±0,0	1,00	3,9±0,3	1,00	3,9±0,3	1,00	3,8±0,4	1,00	3,9±0,2	0,74	0,005
Metodología												
Población diana	1,00	3,8±0,4	0,90	3,8±0,6	1,00	3,9±0,3	1,00	3,9±0,3	0,90	3,8±0,4	0,68	0,001
Recolección de información	1,00	4,0±0,0	1,00	3,9±0,3	1,00	4,0±0,0	0,90	3,7±0,6	0,90	3,9±0,2	0,80	0,007
Recursos didácticos y pedagógicos	1,00	3,8±0,4	1,00	3,8±0,4	1,00	3,8±0,4	0,90	3,6±0,9	0,90	3,7±0,5	0,51	<0,001
Análisis de los resultados de aprendizaje	0,90	3,8±0,6	1,00	3,8±0,4	1,00	3,9±0,3	0,90	3,8±0,6	0,90	3,8±0,4	0,68	0,001
Consideraciones éticas	0,90	3,7±0,6	0,90	3,6±0,3	0,90	3,7±0,6	0,90	3,7±0,6	0,90	3,6±0,6	0,45	0,002
Referencias	1,00	3,8±0,4	1,00	3,9±0,3	1,00	3,8±0,4	1,00	3,9±0,3	1,00	3,8±0,3	0,62	0,002
X±DE	0,96	3,7±0,4	0,95	3,7±0,4	0,98	3,8±0,3	0,93	3,7±0,5	0,96	3,7±0,4		

***Abreviaturas: IVC: Indice de Validez de Contenido;* X*: media aritmética; DE: Desviación Estándar; Concordancia: concordancia entre los jueces, con el coeficiente de Brennan and Prediger.***

## Discusión

El desarrollo de las guías de aprendizaje de la estrategia hacia el cuidado de la vida fue desarrolladas en el marco del proyecto de investigación estrategia educativa para el fortalecimiento de la agencia de autocuidado “Hacia el cuidado de la vida” las cuales incluían el autocuidado mental y espiritual, estilos de vida saludables, autocuidado corporal y cuidado en la interacción social. Estos fueron considerados los contenidos mas relevantes para el fortalecimiento de la agencia de autocuidado en los estudiantes de primer semestre de enfermería. De tal manera, que la construcción de la intervención se basó en una revisión rigurosa de literatura donde la validación por parte de los jueces fue de vital importancia, así como la evaluación por parte de los estudiantes para el anclaje científico y atributos de calidad que se discutirán derivados de este proceso.

Similar al estudio de Galindo et al^21^, en nuestra investigación todos los jueces eran enfermeras y enfermeros con un nivel educativo de posgrado en maestría, experiencia investigatidora, docente y administrativa.

En relación a la validación por parte de los jueces de la intervención educativa hacia el cuidado de la vida se obtuvo un índice de validez de contenido con valores superiores a lo deseable, lo cual es similar a lo reportado por otros autores donde la validación de contenido educativo por jueces obtuvo una valoración de la información como suficiente y necesaria^21^^-^^23^.

Los resultados mencionados apuntan a la pertinencia de la evaluación de los especialistas sobre la calidad y suficiencia del contenido de la intervención propuesta en el presente estudio, similar a los resultados Galdino et al^24^, en su estudio denominado validación de un folleto sobre el autocuidado del pie diabético donde los jueces del área de enfermería permitieron la validación de material con un índice de validez del contenido de 0.99 y adicionalmente al igual que en nuestro estudio el folleto educativo resultó ser un material educativo válido y confiable para ser utilizado en la población objetivo para el cual fue diseñado.

Adicionalmente se obtuvo un adecuado nivel de concordancia de los jueces demostrando una claridad de la información frente a los aspectos de justificación, objetivos, fundamentación teórica, metodología y consideraciones éticas. Lo anterior similar a otros estudios, donde hubo un nivel de acuerdo satisfactorio en la mayoría de los ítems y adecuado nivel de concordancia entre enfermeras y entre la población objeto^21^^,^^25^. La validación de la estrategia educativa basada en aprendizaje en problemas por parte los participantes dejo de manifiesto la postura intra e interpersonal frente al reconocimiento de la importancia del bienestar espiritual y mental como unidad temática de la estrategia educativa para prevenir el suicidio y la depresión. De esto podemos evidenciar de acuerdo a lo manifestado por los estudiantes una vivencia del aprendizaje hacia la propia experiencia de vida personal y familiar de cada uno de los participantes.

Lo anterior demuestra una coherencia lógico metodológica, donde a través del análisis lexicometrico no solo se identificó un entendimiento del autocuidado y las formas de fortalecerlo, sino también una inducción a la acción que apoyo la toma de decisiones a través de la construcción de vivencias del sujeto encarnado, donde comenzamos a pensar en un cuerpo multidimensional: un compuesto material y energético, personal y vinculado, real y virtual^25^. Lo anterior similar a los reportes de un estudio realizado en Brasil, donde se usó el aprendizaje significativo como estrategia metologica y se consideró una perspectiva constructivista como eje fundamental en la enseñanza del soporte vital básico de los participantes^27^.

Finalmente, el equipo investigador manifiesta que el uso de las estrategias educativas contribuye significativamente a los aprendizajes desde identificación, aceptación e inducción hacia la propia experiencia y la trasformación de comportamientos y estilos de vida. Ademas, de tener en cuenta la necesidad de estas intervenciones por las afecciones en salud mental como estrés en estudiantes de enfermería generado por la pandemia de Covid-19^28^.

Esto ha sido evidenciado en algunos estudios donde por ejemplo el uso de las aplicaciones tecnológicas y otras estrategias de aprendizaje como la simulación clínica han contribuido significativamente en los aprendizajes en diferentes escenarios de salud^29^^,^^31^.

## Conclusiones

La estrategia educativa “Hacia el cuidado de la vida” obtuvo resultados de validación de contenido tanto por jueces expertos como de la población objetivo, lo cual permitió evidenciar en esta última, el entendimiento, la identificación con el concepto de autocuidado, la aceptación e inducción hacia la acción manifestado por la reflexión hacia la transformación de algunos comportamientos de autocuidado en los estudiantes de primer semestre de enfermería.
